# Pharmacokinetic and pharmacodynamic effects of comedication of clopidogrel and dabigatran etexilate in healthy male volunteers

**DOI:** 10.1007/s00228-012-1304-8

**Published:** 2012-07-11

**Authors:** Sebastian Härtter, Regina Sennewald, Cornelia Schepers, Sybille Baumann, Holger Fritsch, Jeffrey Friedman

**Affiliations:** 1 Boehringer Ingelheim Pharma GmbH & Co. KG, Translational Medicine, Biberach an der Riss, Germany; 2 Boehringer Ingelheim Pharma GmbH & Co. KG, Medical Data Services, Ingelheim am Rhein, Germany; 3 CRS, Clinical Research Services Mannheim GmbH, Mannheim, Germany; 4 Boehringer Ingelheim Pharmaceuticals Inc, Ridgefield, CT USA

**Keywords:** Clopidogrel, Comedication, Dabigatran etexilate, Pharmacodynamics, Pharmacokinetics, Thrombin inhibitor

## Abstract

**Purpose:**

To evaluate the pharmacokinetic and pharmacodynamic effects of concomitant administration of single loading doses of clopidogrel or multiple doses of clopidogrel with multiple doses of dabigatran etexilate.

**Methods:**

This was an open-label trial in healthy male subjects. In part 1 (pilot, *n* = 8) and part 3 (*n* = 12), a single dose of clopidogrel (300 or 600 mg, respectively) was given concomitantly with dabigatran etexilate at steady state; part 2 was a randomized, multiple-dose, crossover study with the test treatment being clopidogrel at steady state [300 mg loading dose on day 1, then 75 mg once daily (qd)] with concomitant dabigatran.

**Results:**

Bioavailability was moderately increased when a loading dose of clopidogrel (300 mg in part 1 and 600 mg in part 3) was administered concomitantly with dabigatran etexilate 150 mg twice daily (bid). Test/reference ratios for AUC_τ,ss_ were 135% (90% CI 107–169%) and 132% (90% CI 112–156%), respectively. Steady-state dosing of clopidogrel 75 mg qd and dabigatran etexilate 150 mg bid (part 2) demonstrated minor effects on dabigatran pharmacokinetics (AUC_τ,ss_ ratio test/reference: 91.9%, 90% CI 78.7–107%) or its pharmacokinetic/pharmacodynamic relationships (activated partial thromboplastin time, ecarin clotting time, thrombin time). Similarly, clopidogrel bioavailability remained unchanged by chronic administration of dabigatran etexilate (part 3: ratio test/reference for AUC_0−24_ was 103%; 90% CI 80.3–131%), as did its pharmacodynamic effects on the inhibition of platelet aggregation.

**Conclusions:**

When given concomitantly, dabigatran etexilate and clopidogrel at clinically relevant doses did not appear to have significant effects on the pharmacokinetic and pharmacodynamic profiles of either agent.

**Electronic supplementary material:**

The online version of this article (doi:10.1007/s00228-012-1304-8) contains supplementary material, which is available to authorized users.

## Introduction

Anticoagulants and antiplatelet drugs are among the major therapeutic agents in the treatment of coronary artery disease and the treatment and prevention of arterial and venous thromboembolic events. Given the different mechanisms of action of these two agents, combining them has the potential for additive and perhaps synergistic reductions in thromboembolic events but may also lead to increased bleeding [1, 2].

Dabigatran etexilate (Pradaxa®; Boehringer Ingelheim Pharma GmbH & Co. KG, Ingelheim, Germany) is an oral prodrug that is rapidly converted to dabigatran, a direct thrombin inhibitor [3, 4]. Because of thrombin’s multiple roles in coagulation, thrombin inhibitors not only block fibrin formation but also attenuate further thrombin formation and platelet activation [5]. Dabigatran is subject to conjugation with activated glucuronic acid, yielding pharmacologically active glucuronide conjugates that comprise about 20% of the total dabigatran in plasma [6, 7]. Dabigatran is not metabolized by hepatic cytochrome P450 (CYP) isoenzymes and does not affect the metabolism of other drugs that utilize this system. However, the prodrug but not dabigatran is a substrate for the efflux transporter P-gp [8]. Clopidogrel also has been shown to have some affinity for P-gp [9]. Platelet activation can occur via the platelet thromboxane A_2_ pathway, the adenosine diphosphate (ADP) pathway and via phosphodiesterase.

Clopidogrel inhibits the platelet-surface ADP receptor P2Y_12_, decreasing platelet activation and aggregation [10]. Clopidogrel, also a prodrug, has no intrinsic antiplatelet activity and requires hepatic CYP metabolic activation to produce its pharmacologically active metabolite, which irreversibly inhibits ADP receptors on platelets [10, 11]. Multiple CYP enzymes have been implicated, although CYP2C19 and CYP3A4 are more important in the formation of the pharmacologically active metabolite [12, 13]. Polymorphisms in CYP enzymes may contribute to the high variability in responsiveness to clopidogrel. Studies suggest that a high loading dose of clopidogrel may reduce intra-individual variability and enhance responsiveness [14–17], hence use of a clopidogrel 600 mg loading dose is widespread in clinical practice.

Concomitant use of anticoagulants and antiplatelet therapy is frequent in clinical practice [1, 2]. Atrial fibrillation patients on anticoagulant therapy for stroke prevention commonly have concomitant conditions such as coronary heart disease and cerebrovascular disease, for which antiplatelet drugs are prescribed. In these patients concerns about increased bleeding risks exist [18]. Accordingly, the potential for increased risk of bleeding when the combination of an anticoagulant and antiplatelet drug is taken is addressed in the label of dabigatran etexilate [8]. This study was conducted to evaluate whether any pharmacokinetic (PK) and/or pharmacodynamic (PD) interactions exist.

The objectives of this study were to evaluate (1) the PK and PD effects of concomitant administration of multiple doses of clopidogrel 75 mg once daily (qd) (preceded by a loading dose of clopidogrel 300 mg) with multiple doses of dabigatran etexilate 150 mg twice daily (bid) and (2) the PK and PD effects of concomitant administration of single loading doses of clopidogrel (300 or 600 mg) with dabigatran etexilate (150 mg bid). Secondary PD parameters evaluated were activated partial thromboplastin time (aPTT), ecarin clotting time (ECT), thrombin time (TT), inhibition of platelet aggregation as well as effects on capillary bleeding time (CBT).

## Methods

### Subjects

Eligible subjects were healthy males aged 18–40 years, with a body mass index (BMI) of 18.5–29.9 kg/m^2^, and judged to be healthy based on medical history, physical examination, vital signs, 12-lead electrocardiogram (ECG) and clinical laboratory tests. Subjects were excluded if they had any of the following: gastrointestinal, hepatic, renal, respiratory, cardiovascular, metabolic, immunological, or hormonal disorders; trauma or surgery within the last month or planned surgery during the trial; risk of bleeding; use of drugs that might affect blood clotting; or use of drugs that might influence the results of the trial (e.g., inhibitors or inducers of P-gp, CYP3A4, CYP2C9, or CYP2C19). All eligible subjects signed written informed consent prior to enrolment. All subjects were genotyped for single nucleotide polymorphisms in the *CYP2C19* and *CYP2C9* genes.

### Study design and treatments

This open-label drug–drug interaction trial in healthy male subjects was conducted in a single center and included a screening evaluation prior to the conduct of any study procedures and an end-of-study evaluation for all subjects. Prior to study initiation, the trial was reviewed and approved by an independent ethics committee (Ethik-Kommission der Landesärztekammer Baden-Württemberg in Stuttgart, Germany) and competent authority (BfArM, Germany). The year of approval and trial conduct was 2009. The trial was registered as required according to German drug law.

The treatment phase was divided into three separate parts, each with a different set of subjects. Part 1 (pilot study) was an open-label, multiple-dose (for dabigatran etexilate) and single-dose (for clopidogrel), fixed-sequence study. A single dose of clopidogrel 300 mg was given in addition to dabigatran etexilate 150 mg bid in steady state (Table 1). Part 2 (main study) was a randomized, open-label, multiple-dose, crossover study, with three periods of up to 5 days separated by washout periods of at least 14 days. The test treatment consisted of a clopidogrel 300 mg loading dose on day 1, followed by a 75 mg dose on day 2, then clopidogrel 75 mg qd in combination with dabigatran etexilate 150 mg bid on days 3–5 (Table 1). Part 3 (main study) was an open-label, multiple-dose (for dabigatran etexilate) and single-dose (for clopidogrel), fixed-sequence study. A single dose of clopidogrel 600 mg was given alone or in addition to dabigatran 150 mg bid at steady state (Table 1).

**Table 1:**
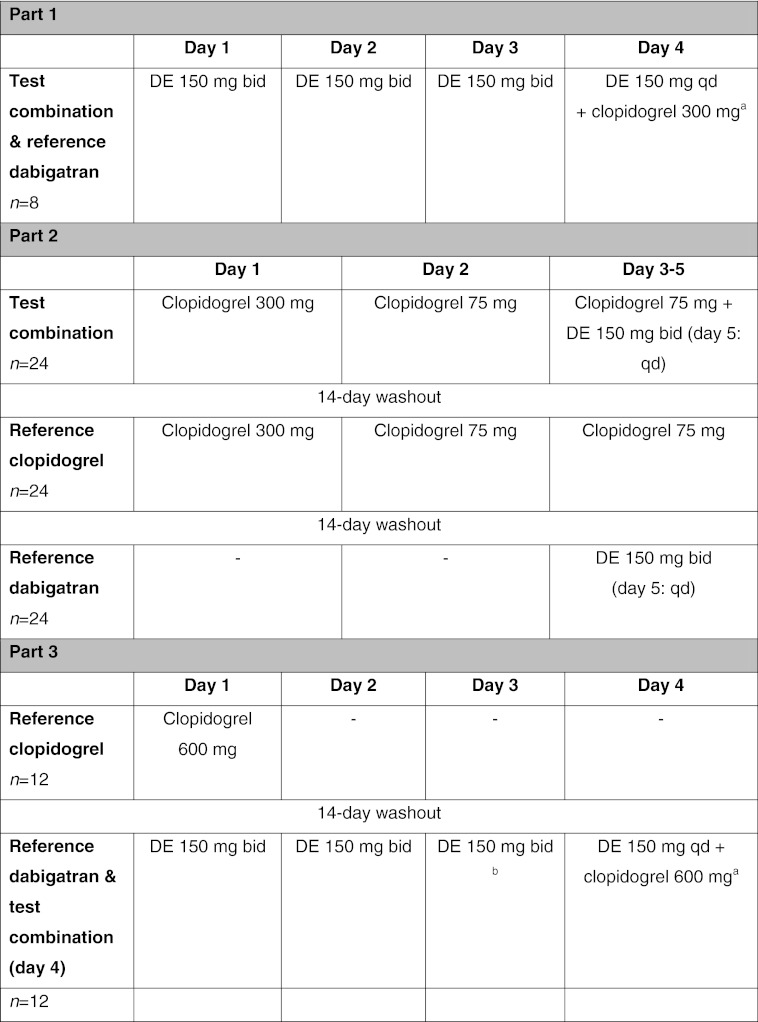
Study design

*DE* dabigatran etexilate, *bid* twice daily, *qd* once daily

^a^Test dabigatran pharmacokinetic parameters determined from samples taken after the co-administered doses on day 4

^b^Reference dabigatran pharmacokinetic parameters determined from samples taken after the morning dose on day 3

Although there are no reported interactions of food with dabigatran etexilate or clopidogrel [8, 19], the morning medication was administered with about 240 mL of water following an overnight fast of at least 10 h, and no food was allowed until 4 h after drug administration. According to the twice-daily regimen dabigatran etexilate was similarly administered again 12 h later. Subjects were not allowed to lie down during the 2 h following drug administration, and water was limited for 1 h before and after drug administration. On dosing days a light non-standardized breakfast was given 1 h after morning drug administration. On profile days, no food was given until 4 h after administration. No concomitant therapy was allowed during the study, except for prophylactic thyroid hormone replacement and for symptomatic therapy of adverse events (AEs) in need of treatment.

### Study endpoints

The primary endpoints for PK were the maximum measured plasma concentration of the analyte at steady state (C_max,ss_) and the area under the concentration–time curve over one dosing interval (0–12 h for dabigatran and 0–24 h for clopidogrel) at steady state (AUC_τ,ss_) for free and total dabigatran (free and glucuronide-conjugated) and for clopidogrel and its inactive metabolite (SR26334), and the C_max_ and the AUC from 0 to 24 h (AUC_0–24_) of clopidogrel and SR26334 after the 300 or 600 mg clopidogrel loading doses.

The primary endpoints for PD were area under the effect curve of ADP-induced inhibition of platelet aggregation (IPA) at steady state of clopidogrel over one dosing interval from 0–24 h after dosing (AUEC_IPA,τ,ss_); maximum percentage change compared with baseline in ADP-induced platelet aggregation at steady state of clopidogrel (E_max,IPA,ss_); AUEC from 0 to 24 h (AUEC_IPA,0–24_) after the loading dose of clopidogrel 600 mg; and the maximum percentage change compared with baseline in ADP-induced platelet aggregation from 0 to 24 h (E_max,IPA,0–24_) after the loading dose of clopidogrel 600 mg.

The secondary endpoints included other PK parameters and the effects of clopidogrel on the PD of dabigatran assessed by ECT, aPTT, TT (including maximum effect ratio, ER_max_) as well as the CBT.

### PK/PD determinations (bioanalytics)

For PK parameter evaluations, blood samples were taken for the determination of plasma concentrations of free, nonconjugated dabigatran, total dabigatran (after alkaline cleavage of conjugates), and the prodrug dabigatran etexilate, and of clopidogrel and SR26334.

The following PD parameters were also evaluated:

- For clopidogrel, ADP-induced IPA was determined at predefined time points.
- Capillary bleeding times were assessed (part 2 and part 3 of the study) before clopidogrel administration, 2, 4, and 12 h after clopidogrel administration, and following administration of dabigatran etexilate alone.

Blood samples were taken before and at specified intervals up to 24 h after the morning clopidogrel dose, and before and up to 24 h after the morning and evening dabigatran etexilate dose. Timings of PK/PD sampling are shown in Supplementary Table 1.

Blood samples (∼2.7 mL for dabigatran and ∼4.0 mL for clopidogrel) were collected into tubes containing potassium ethylenediaminetetraacetic acid for PK analysis. Samples were centrifuged, and the resulting plasma samples were separated and stored at −70°C or below.

Plasma concentrations of free, nonconjugated dabigatran; total dabigatran (sum of free and conjugated dabigatran measured after alkaline cleavage of conjugates); the prodrug dabigatran etexilate; clopidogrel; and SR26334 were determined by validated high-performance liquid chromatography tandem mass spectrometry (HPLC-MS/MS) at AAI Pharma Deutschland (Neu-Ulm, Germany). The calibration curves for free and total dabigatran covered a range from 1.00 to 400 ng/mL plasma in undiluted samples. The calibration curves were linear over the calibration range and no interference from endogenous compounds was observed. Maximum imprecision and inaccuracy for quality control samples at concentrations of 3, 30, and 320 ng/mL were 6.62 and +5.86%, respectively. For the prodrug, maximum imprecision and inaccuracy were 6.63 and +2.83%, respectively.

Calibration curves for clopidogrel and SR26334 covered concentration ranges of 0.01–10 ng/mL and 0.02–20 μg/mL, respectively, with an inaccuracy generally below 4% and imprecision below 9%. For blood coagulation parameters aPTT, ECT, and TT (PD analyses), ∼3 mL of blood were collected in sodium citrate monovettes at the time points indicated for PK analyses. The blood samples were centrifuged, frozen, and stored at −20°C or below. The aPTT, ECT, and TT parameters were analyzed on an MC10PLUS coagulometer using validated methods by BiochemA, Riegel, Germany. aPTT was measured using a kit from Roche Diagnostics (Mannheim, Germany) according to manufacturer’s instructions, and the ECT assay utilized ecarin from Pentapharm (Basel, Switzerland). Thrombin inhibition (TT), anti-factor IIa, was measured by the Hemoclot Thrombin Inhibitors Assay® (Hemoclot® TT #CK002K, HYPHEN BioMed, Neuville-sur-Oise, France) according to the manufacturer’s instructions. The Hemoclot kit was used for the quantitative measurement of direct thrombin inhibitors in plasma, with a clotting method based on the inhibition of a constant and defined concentration of thrombin.

For platelet aggregation testing, ∼9 mL of blood was collected into a 10 mL monovette containing citrate to yield a final concentration of 10.6 mM. Platelet-rich plasma (PRP) was prepared by centrifugation and removed from the red-cell sediment. Approximately 0.5 mL platelet-poor plasma (PPP) was prepared from PRP by further centrifugation. Inhibition of platelet aggregation was performed by light transmission aggregometry in citrated PRP, using an 8-channel platelet aggregation profiler (PAP 8) aggregometer (möLab, Germany). The PRP (0% light transmission) and PPP (100% light transmission) served as reference. After baseline adjustment, ADP in a final concentration of 20 μM was added and aggregation recorded for 6 min. Maximum aggregation (%) within the first 6 min after addition of ADP was determined.

The bleeding time was assessed by single measurements on the forearm using the Surgicutt® Bleeding Time Device (Product Number SUB50, International Technidyne, Edison, NJ, USA). Using a Surgicutt device, a standardized incision of 5 mm length and 1 mm depth was made in the skin. Every 30 s, the blood flow was wicked away cautiously with filter paper, so that the platelet plug remained intact, until the bleeding stopped. Normal bleeding time was under 8 min.

### Safety

Safety was assessed by medical examination, pulse rate, blood pressure, ECG, clinical and laboratory parameters, AEs, and an assessment of clinical global tolerability (4-point scale: good, satisfactory, not satisfactory, and bad).

### Statistical methods

The aim of the trial was to estimate the relative bioavailability (ratio of exposure: test/reference) and its 90% confidence interval (CI) regarding PK and PD parameters (as listed below). Different test and reference treatments were compared by applying the average bioequivalence method. Point estimates for the test/reference ratios and the corresponding 90% CIs were calculated using an analysis of variance (ANOVA) based on the logarithmic scale regarding PK parameters (multiplicative model) and on the original scale for PD parameters (additive model). The ANOVA model included effects accounting for the following sources of variation: “sequence,” “subject within sequence,” “period,” and “treatment” (crossover design of part 2). The effect “subject within sequence” was considered as random, whereas the other effects were considered as fixed. For parts 1 and 3 “sequence” was not considered because of the fixed sequence design. Two-sided 90% CIs for the test/reference ratios were interpreted exploratively, and no statistical hypotheses were tested in a confirmatory sense.

In pilot part 1 and main part 3, the following primary PK and PD parameters were compared intra-individually:

- AUC_τ,ss_ and C_max,ss_ of total dabigatran
- C_max_ and the AUC from 0 to 24 h (AUC_0–24_) of clopidogrel and inactive metabolite SR26334
- AUEC_IPA,0–24_ and E_max,IPA,0–24_

In main part 2, the following primary PK or PD parameters were compared intra-individually:

- AUC_τ,ss_ and C_max,ss_ of total dabigatran, clopidogrel, and inactive metabolite SR26334
- AUEC_IPA,τ,ss_ and E_max,IPA,ss_ (syn. to AUEC_IPA,0–24_ and E_max,IPA,0–24_ at steady state)

AUEC and ER_max_ of aPTT, ECT, and TT as well as CBT, and safety and tolerability were assessed descriptively as secondary parameters.

For the CBT individual listings including the baseline value, the maximum value of the post-dose measurements, and the corresponding time of the maximum value were provided. Frequency tables of the three categories (<10, 10–30, >30 min) for the maximum CBT values were provided (part 2 and part 3).

Sample sizes of 8 subjects in part 1 and 12 subjects in part 3 of the trial were considered sufficient to achieve the exploratory aims. The sample size of 24 subjects in part 2 was chosen to ensure that the estimation of ratios is precise. The desired precision is represented by the half-width of the 90% confidence interval of the point estimate on the logarithmic scale. In previous single-dose studies, intra-individual variabilities (gCVs) ranging from about 40 to 50% were observed for PK exposure parameters. The gCV after multiple dosing can be expected to be lower. In case of a gCV of 35%, a 90% CI for the ratio of the test/reference means for a PK parameter would be obtained with 95% probability and a desired precision of 0.2231 (on the logarithmic scale). For a ratio of 100%, this precision corresponds to an interval of 80 to 125%.

The calculation was performed using the MOC3 routine from commercial software nQuery Advisor [20].

## Results

### Study subjects (parts 1, 2, and 3)

A total of 44 healthy male volunteers entered the study and were treated: 8 subjects in part 1, 24 in part 2, and 12 in part 3. A summary of demographic and baseline characteristics is shown in Table 2. All subjects in the trial were white, except for one who was of American-Indian/Alaska native ethnic origin. Genotyping revealed that three subjects were homozygous for the deficient *2 allele of *CYP2C19*, nine were heterozygous carriers of the CYP2C19 *2 allele, four subjects had the CYP2C19 *17 allele (leading to higher CYP2C19 activity), and two subjects carried only low-activity alleles *2 or *3 of *CYP2C9*, whereas 11 showed the CYP2C9*1/*2 or *1/*3 genotype. All other subjects were genotyped as normal (extensive) metabolizers. Forty subjects completed the trial.

**Table 2:** Demographic and other baseline characteristics

	Part 1 (*n* = 8)	Part 2 (*n* = 24)	Part 3 (*n* = 12)
Age, years, mean ± SD (range)	28.9 ± 4.1 (22–33)	31.5 ± 5.5 (23–40)	32.8 ± 5.6 (22–40)
Ethnicity, *n* (%)			
White	8 (100.0)	24 (100.0)	11 (91.7)
American Indian/Alaska native	0	0	1 (8.3)
Weight, kg, mean ± SD (range)	79.6 ± 9.9 (69–96)	80.4 ± 7.4 (64–91)	81.6 ± 9.4 (68–100)
BMI, kg/m^2^, mean ± SD (range)	24.89 ± 2.52 (21.8–29.4)	24.92 ± 2.32 (19.3–28.7)	26.45 ± 1.73 (24.1–29.5)
Smokers, *n* (%)			
Never smoked	3 (37.5)	15 (62.5)	5 (41.7)
Ex-smoker	2 (25.0)	8 (33.3)	1 (8.3)
Current smoker	3 (37.5)	1 (4.2)	6 (50.0)
Alcohol intake, *n* (%)			
Non-drinker	4 (50.0)	13 (54.2)	1 (8.3)
Drinker, no interference	4 (50.0)	11 (45.8)	11 (91.7)

*BMI* Body mass index, *SD* standard deviation

### Part I

#### PK parameters

Maximum plasma concentrations of free and total dabigatran were increased when clopidogrel 300 mg was coadministered with dabigatran etexilate 150 mg compared with concentrations observed when dabigatran etexilate was administered alone. The shapes of the plasma-concentration-time curves of free and total dabigatran and clopidogrel and SR26334 were similar with or without coadministration of 300 mg clopidogrel. C_max,ss_ increased by 69% when clopidogrel 300 mg was coadministered with dabigatran 150 mg bid. The corresponding change in AUC_τ,ss_ of total dabigatran was a 35% increase when clopidogrel 300 mg was coadministered with dabigatran 150 mg bid (Table 3). Clopidogrel was not associated with other changes in total dabigatran PK parameters.

**Table 3:** Part 1. Adjusted geometric means (gMean) and gMean ratios of total dabigatran pharmacokinetic parameters when dabigatran etexilate 150 mg was administered twice daily with or without a single dose of clopidogrel 300 mg

Parameter	DE 150 mg + clopidogrel 300 mg	DE 150 mg	Ratio test/reference, % gMean	90% Cl	Intra-individual gCV, %
	Test, gMean (*n* = 8)	Reference, gMean (*n* = 8)			
Total dabigatran					
AUC_τ,ss_, ng·h/mL	979	727	135	107, 169	24.4
C_max,ss_, ng/mL	184	109	169	135, 212	24.0
CL/F_ss_, mL/min	1920	2580	74.4		

*AUC*_*τ,ss*_ Area under the concentration–time curve of the analyte in plasma at steady state over a uniform dosing interval τ, *CI* confidence interval, *CL/F*_*ss*_ apparent clearance of analyte in plasma following extravascular administration, *C*_*max,*_ _*ss*_ maximum plasma concentration at steady state, *DE* dabigatran etexilate, *gCV* geometric coefficient of variation

AUC_0–24_ for clopidogrel with dabigatran etexilate 150 mg bid was 8,580 pg·h/mL and for SR 26334 with dabigatran etexilate 150 mg bid was 35,400 pg h/mL. Large variability [geometric coefficient of variation (gCV)] between 171 and 258% with respect to the primary PK parameters of clopidogrel was observed, although the variability for the major circulating metabolite SR26334 was low (13–21% gCV).

#### PD parameters

##### Inhibition of platelet aggregation

A maximum of about 50% inhibition of ADP-dependent platelet aggregation (E_max_) was observed after a single dose of clopidogrel 300 mg (part 1; data not shown).

##### Blood coagulation parameters (aPTT, ECT, and anti-factor IIa)

In part 1, the effect of dabigatran on these coagulation parameters was slightly increased when a single dose of clopidogrel 300 mg was added to steady-state dabigatran etexilate 150 mg bid. The greatest change was seen in the gMean AUEC_τ,ss_ of aPTT, which was increased by about 30% when dabigatran etexilate was administered with clopidogrel.

### Part 2

#### PK parameters

There was no apparent change in the shape of the plasma concentration–time curves of dabigatran in subjects who received dabigatran etexilate 150 mg alone or after multiple doses of clopidogrel 75 mg qd (preceded by a loading dose of 300 mg). Similarly, dabigatran etexilate 150 mg bid had no influence on the shape of the plasma concentration–time curves of clopidogrel and SR26334 after multiple doses of clopidogrel (Supplementary Fig. 1a, b).

Repeated doses of clopidogrel had no effect on the steady-state PK (AUC_τ,ss_ and C_max,ss_) of free and total dabigatran, and intra-individual variations were similar in each case. Clopidogrel had no effect on dabigatran etexilate glucuronidation. Table 4 shows the ratios and confidence intervals of the PK parameters of dabigatran etexilate 150 mg bid when given in combination with clopidogrel 75 mg qd (preceded by a 300 mg loading dose) versus dabigatran 150 mg alone.

**Table 4:**
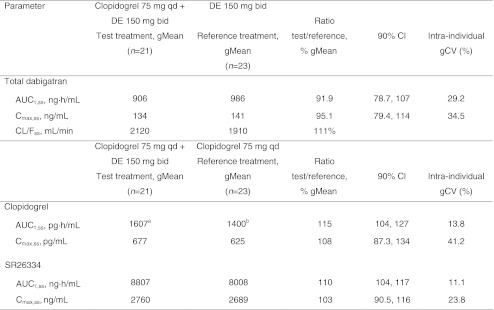
Part 2. Adjusted geometric means (gMean) and gMean ratios of total dabigatran, clopidogrel, and SR26334 multiple dose pharmacokinetic parameters when dabigatran etexilate (DE) 150 mg twice daily (bid) was administered in combination with clopidogrel 75 mg once daily (qd) (preceded by a 300 mg loading dose) versus dabigatran etexilate 150 mg bid alone or clopidogrel 75 mg qd alone

*AUC*_*τ,ss*_ Area under the concentration–time curve of the analyte in plasma at steady state over a uniform dosing interval τ, *CI* confidence interval, *CL/F*_*ss*_ apparent clearance of analyte in plasma following extravascular administration, *C*_*max,*_ _*ss*_ maximum plasma concentration at steady state, *gCV* geometric coefficient of variation

^a^ *n* = 14

^b^ *n* = 17

The corresponding ratios and confidence intervals of the PK parameters of clopidogrel and SR26334 with dabigatran etexilate 150 mg bid given in combination with clopidogrel 75 mg qd (preceded by a 300 mg loading dose) versus clopidogrel 75 mg alone showed that concomitant administration did not alter exposure to the parent drug or the major metabolite.

The time to C_max_ (t_max,ss_; 2 h) and half-life (t_1/2,ss_; ∼9.5 h) were similar in subjects receiving multiple doses of dabigatran etexilate 150 mg alone or in combination with repeated oral doses of clopidogrel 75 mg (300 mg loading dose).

#### PD parameters

##### Inhibition of platelet aggregation

**Fig. 1a, b:**
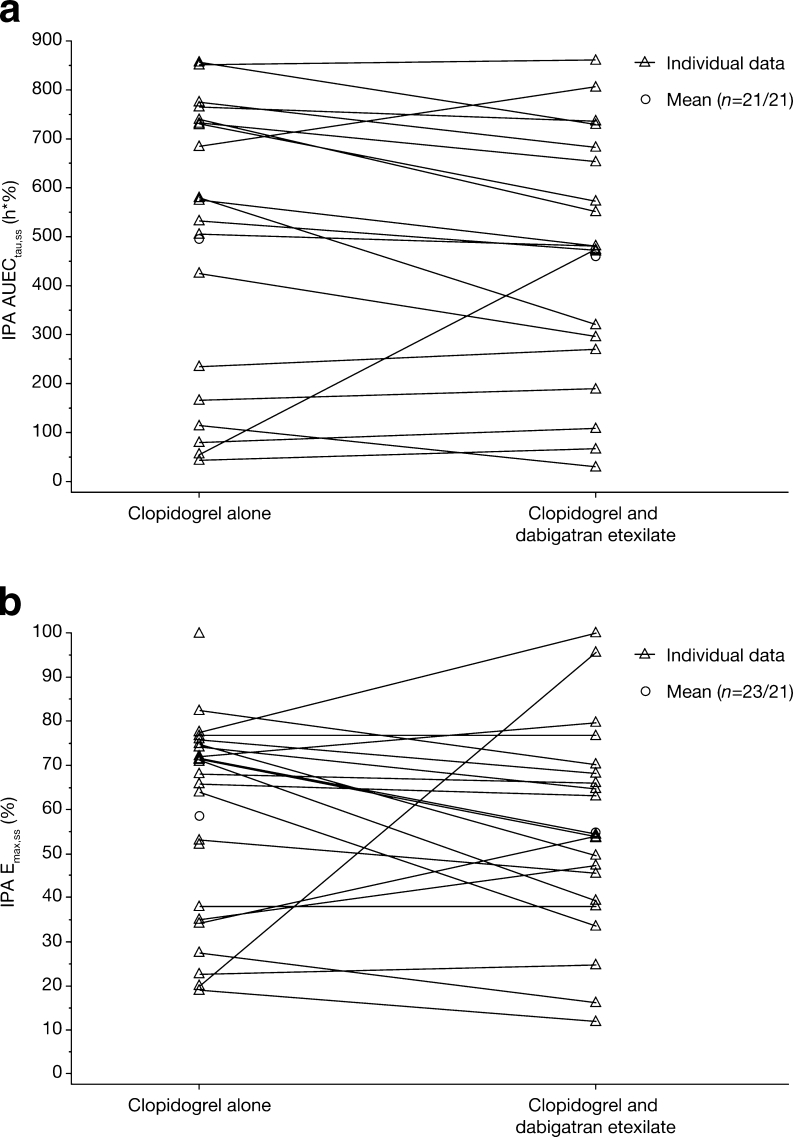
Part 2. Effect of comedication with dabigatran on inhibition of platelet aggregation (IPA) by clopidogrel. Comparison between clopidogrel monotherapy and clopidogrel + dabigatran of individual and geometric mean (gMean) AUEC_τ,ss_ (**a**) and E_max,ss_ values of IPA (**b**). *AUEC*_*τ,ss*_ Area under the effect curve of inhibition of platelet aggregation (at steady state), *E*_*max,ss*_ maximum percentage change (compared with baseline) in adenosine triphosphate-induced platelet aggregation of clopidogrel (at steady state)

Coadministration of dabigatran etexilate 150 mg did not alter the maximum ADP-dependent platelet aggregation achieved after a single dose of clopidogrel 300 mg (Fig. 1). Following repeated doses of clopidogrel 75 mg (preceded by 300 mg loading dose), the E_max,ss_ of IPA was approximately 50% with or without coadministration of multiple doses of dabigatran etexilate 150 mg bid (part 2).

Analyses revealed modestly lower AUEC_τ,__ss_ and E_max,ss_ of IPA with regard to the comparison of the combined treatment with multiple doses of clopidogrel 75 mg qd (preceded by a loading dose of 300 mg) and multiple doses of dabigatran etexilate 150 mg versus treatment with multiple doses of clopidogrel alone (Table 6).

##### Blood coagulation parameters (aPTT, ECT and anti-factor IIa)

In part 2, AUEC_IPA,_ _τ,ss_ and ER_max,ss_ of TT, ECT, and aPTT were not altered after concomitant administration of repeated doses of clopidogrel 75 mg qd (300 mg loading dose) and multiple doses of dabigatran etexilate 150 mg bid. A trend towards a higher IPA activity was observed in subjects with the CYP2C19*17 allele. CYP2C19*17 is associated with faster metabolism of clopidogrel to its pharmacologically active metabolite [21]. *CYP2C9* polymorphism was not associated with differences in maximum IPA activities.

**Fig. 2a–c:**
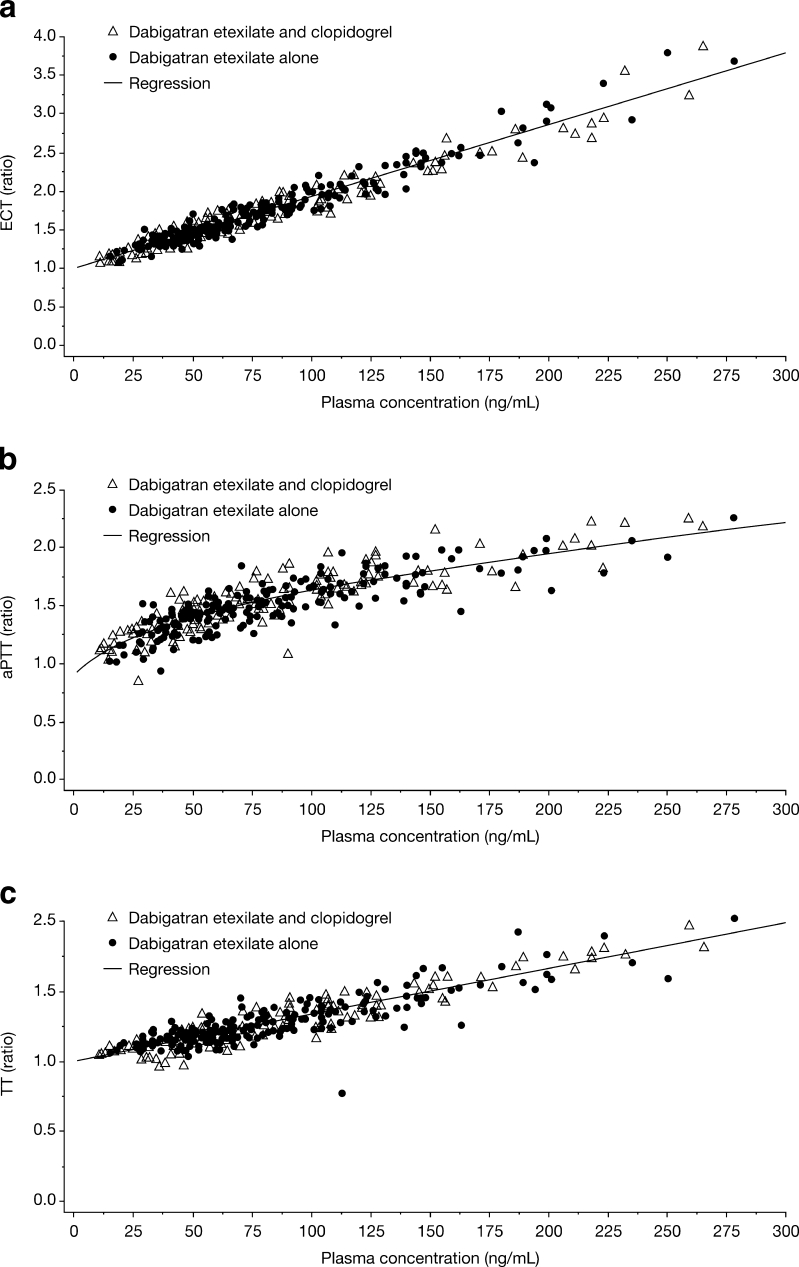
Part 2. Effect of clopidogrel on ecarin clotting time (ECT) ratio (**a**), activated partial thromboplastin time (aPTT) ratio (**b**), and TT (anti-factor IIa) ratio (**c**) after multiple oral administrations of dabigatran etexilate 150 mg twice daily with or without coadministration of repeated doses of 75 mg clopidogrel once daily (300 mg loading dose)

In evaluation of PK/PD, a linear relationship between plasma concentration and coagulation prolongation was observed for both TT and ECT, whereas the relationship was nonlinear between plasma concentration and aPTT. No effect of clopidogrel on the PK/PD relationship was found when dabigatran etexilate was given alone or in combination with clopidogrel (Fig. 2).

##### Capillary bleeding time

In part 2, the maximum CBT was prolonged by any treatment that included clopidogrel. However, no difference could be observed between clopidogrel monotherapy and clopidogrel + dabigatran etexilate combination therapy. Dabigatran alone did not prolong the CBT.

### Part 3

#### PK parameters

The maximum plasma concentrations of free and total dabigatran were increased when clopidogrel 600 mg was coadministered with dabigatran etexilate 150 mg bid at steady state. However, the general shape of the plasma concentration–time profiles of free and total dabigatran was not altered. There was no detectable difference between the plasma concentration–time profiles of clopidogrel or SR26334 when clopidogrel 600 mg was administered alone or with dabigatran etexilate 150 mg bid. Clopidogrel had no effect on dabigatran glucuronidation (Supplementary Fig. 2a, b).

Calculation of geometric mean (gMean) ratios showed that the bioavailability (AUC_τ,ss_) of total dabigatran was increased by 32% when dabigatran etexilate 150 mg bid was administered together with a single dose of clopidogrel 600 mg. The maximum plasma concentration (C_max,ss_) of total dabigatran was similarly affected, increasing by 43% (Table 5). The effect of clopidogrel on the exposure of free dabigatran was similar to that observed with total dabigatran. The AUC_0–24_ and C_max_ for clopidogrel and SR26334 after clopidogrel 600 mg alone were only minimally affected by coadministration with dabigatran 150 mg bid at steady state (Table 5).

**Table 5:**
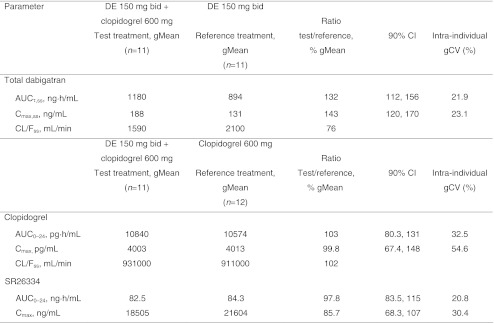
Part 3. Adjusted geometric means (gMean) and gMean ratios of total dabigatran, clopidogrel, and SR26334 pharmacokinetic parameters when dabigatran etexilate (DE) 150 mg twice daily (bid) was administered in combination with a single dose of clopidogrel 600 mg versus dabigatran 150 mg bid alone or clopidogrel 600 mg alone

*AUC*_*τ,ss*_ Area under the concentration–time curve of the analyte in plasma at steady state over a uniform dosing interval τ, *CI* confidence interval, *C*_*max,*_ _*ss*_ maximum plasma concentration at steady state, *CL/F*_*ss*_ apparent clearance of analyte in plasma following extravascular administration, *gCV* geometric coefficient of variation

#### PD parameters

##### Inhibition of platelet aggregation

A maximum of about 60% inhibition of ADP-dependent platelet aggregation (E_max_) was observed after a single dose of clopidogrel 600 mg (part 3; Table 6). The AUEC_τ,__ss_ in participants taking multiple doses of dabigatran etexilate 150 mg concomitantly with a single dose of clopidogrel 600 mg was lower than that of the treatment group taking a single dose of clopidogrel 600 mg (1,179 versus 1,264, respectively; 90% CI –293 to 124) (Table 6).

**Table 6:**
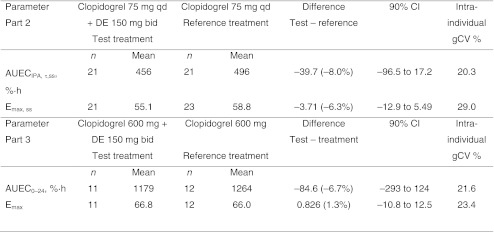
Parts 2 and 3. Means (original scale) and differences in inhibition of ADP-induced platelet aggregation by clopidogrel in combination with dabigatran etexilate (DE) and alone

*AUEC*_*0–24*_ Area under the effect curve of inhibition of platelet aggregation after the loading dose of 600 mg clopidogrel from drug administration until 24 h thereafter (at steady state), *AUEC*_*τ,ss*_ area under the effect curve of inhibition of platelet aggregation (at steady state), *bid* twice daily, *CI* confidence interval, *E*_*max*_ maximum percentage change (compared with baseline) in ADP-induced platelet aggregation of clopidogrel (E_max,ss_, at steady state), *qd* once daily

##### Blood coagulation parameters (aPTT, ECT, and anti-factor IIa)

In part 3, the dabigatran-related effect on TT, ECT, and aPTT after administration of dabigatran etexilate 150 mg bid was slightly increased when a single dose of clopidogrel 600 mg was coadministered. The greatest change (approximately 36.5%) was seen in the AUEC_τ,ss_ of aPTT, when dabigatran etexilate was administered with clopidogrel.

##### Capillary bleeding time

In part 3, the coadministration of dabigatran etexilate 150 mg bid with clopidogrel 600 mg did not increase the CBT compared with a single dose of clopidogrel 600 mg given alone.

### Safety (parts 1, 2, and 3)

All AEs were of mild or moderate intensity. All events resolved by the end of the trial, except for an ongoing condition of hemorrhoids in one subject in part 2. There were no deaths, serious AEs, or AEs of severe intensity.

The most frequently reported AEs in all study parts were nervous system disorders, specifically headache. Four subjects reported AEs considered related to the study drugs. These were hematoma (part 2: one subject, clopidogrel 75 mg; one subject, during washout after the first treatment period), hematuria (part 2: one subject, clopidogrel + dabigatran etexilate), and rash, i.e., exanthema on both hands (part 3: one subject, dabigatran etexilate + clopidogrel).

Overall, four subjects discontinued treatment prematurely. In part 2, two subjects discontinued treatment because of an AE: one for moderate hemorrhoids after receiving dabigatran etexilate alone (considered not to be drug related), and one for moderate hematuria while receiving clopidogrel + dabigatran etexilate. A third subject withdrew his consent after receiving clopidogrel alone. In part 3, one subject withdrew his consent after receiving clopidogrel alone.

Vital signs, ECG, and safety laboratory assessments did not indicate any relevant, consistent, or treatment-related untoward reactions. Global clinical tolerability was good in all subjects, except in one subject during treatment with clopidogrel + dabigatran. This subject, who reported global tolerability as “not satisfactory,” discontinued the trial because of an AE (moderate hematuria).

## Discussion

Given the different mechanisms of action of anticoagulants and antiplatelet drugs, combining these agents has the potential to reduce thromboembolic events and their consequences, although with the likelihood of an increased risk of bleeding [1, 2]. Furthermore, if any drug–drug interactions between the selected anticoagulant and antiplatelet drugs are present, the bleeding risk could be substantially increased. Our study shows that after multiple dosing of clopidogrel 75 mg qd (preceded by a loading dose of 300 mg) plus dabigatran etexilate 150 mg bid, exposure to free and total (free and glucuronide-conjugated) dabigatran remained essentially unchanged compared with dabigatran etexilate monotherapy. Concomitant intake of clopidogrel did not affect dabigatran glucuronidation, and no or only minimal effects of dabigatran on the PK of clopidogrel or its inactive metabolite (SR26334), or on the inhibition of platelet aggregation induced by the active metabolite of clopidogrel were observed.

Clopidogrel in this therapeutic regimen (a 300 mg loading dose followed by 75 mg qd) had only minimal effects on the coagulation markers aPTT, ECT, and TT; the latter two are closely related to thrombin inhibition. Accordingly, the effect of dabigatran and the PK/PD relationship were essentially the same when dabigatran etexilate was given with or without concomitant clopidogrel. A high loading dose of clopidogrel 600 mg also had no effect on the thrombin-related coagulation markers.

Clopidogrel inhibits the platelet-surface ADP receptor P2Y_12_, decreasing platelet activation and aggregation processes [10]. Dabigatran did not influence the inhibition of ADP-dependent platelet aggregation by clopidogrel. Furthermore, dabigatran alone did not prolong CBT, a measure to assess platelet-driven coagulation [22, 23]. Maximum CBT was clearly prolonged by any treatment regimen that included clopidogrel compared with dabigatran monotherapy. However, there was no difference in CBT between clopidogrel given alone or in combination with dabigatran etexilate. In atrial fibrillation patients, however, from the RE-LY trial, concomitant intake of clopidogrel and dabigatran etexilate led to an increased rate of major and any bleeding [24]. In a phase I trial, the first oral direct thrombin inhibitor, ximelagatran, in combination with acetylsalicylic acid (ASA) further prolonged CBT by about 40% compared with ASA alone [25]. Bleeding time with the combination rivaroxaban and clopidogrel was significantly prolonged in four subjects, compared with either drug alone, which increased the overall least square means to 3.77 (90% CI 2.82–4.73) times baseline with the combination, compared with 1.13 times baseline (90% CI 0.17–2.09) with rivaroxaban and 1.96 times baseline (90% CI 0.10– 2.91) with clopidogrel [26]. The results with dabigatran observed in this study seem to some extent different from previously observed data with other oral anticoagulants such as ximelagatran and rivaroxaban.

A high loading dose of clopidogrel 600 mg was associated with an increase (32%) in the bioavailability of dabigatran. Exposure to clopidogrel and SR26334 was similar when clopidogrel was administered alone or with multiple doses of dabigatran etexilate 150 mg bid. The efflux transporter P-gp in the gastrointestinal tract can directly limit oral drug absorption [27, 28]; dabigatran etexilate, but not dabigatran, is a substrate for P-gp [8, 29]. Concomitant administration of strong P-gp inhibitors, including quinidine, amiodarone, verapamil, ketoconazole, and clarithromycin, raises dabigatran serum concentrations [8, 29, 30]. Clopidogrel also has been shown to be a substrate of P-gp [9]. Clopidogrel absorption, and thereby formation of its active metabolite, is diminished by P-gp-mediated efflux [9]. A high loading dose of clopidogrel (either 300 or 600 mg) may have the potential to competitively inhibit intestinal P-gp, causing an increase in dabigatran bioavailability. A high, 600 mg, loading dose of clopidogrel is widely used in clinical practice, especially in acute settings, to reduce intra-individual variability and enhance responsiveness [14–17]. However, this clopidogrel loading dose is given for only 1 day. This transient increase in dabigatran exposure for a single day by about 30% should be taken into consideration particularly in vulnerable patients at high risk of bleeding. However, it remains to be determined whether this effect, which is only observed for the first day of a combination treatment, is likely to have any clinical impact at all.

Unlike dabigatran, which is not metabolized via CYP isoenzymes, clopidogrel requires hepatic CYP metabolic activation to produce its pharmacologically active metabolite [10, 11]. In addition to CYP3A4, CYP2C19 is particularly important relative to other CYP enzymes in the formation of the active metabolite of clopidogrel [12, 13]. As neither dabigatran nor its prodrug is an inhibitor of any CYP, dabigatran comedication should not diminish the therapeutic efficiency of clopidogrel. In accordance with published data [21], subjects carrying the ultrarapid metabolizer allele CYP2C19*17 had a trend to more efficient inhibition of ADP-dependent platelet aggregation. However, in general the *CYP2C19* genotype did not influence PK and PD after comedication with dabigatran etexilate.

Concomitant administration of dabigatran and clopidogrel did not reveal any unexpected safety findings in the healthy male subjects included in this clinical trial. All AEs observed were of mild to moderate intensity, none were serious, and all had resolved by the end of the study, with the exception of ongoing hemorrhoids in one subject. Reported AEs, including hematoma, hematuria, and rash, were consistent with the labelling of dabigatran and clopidogrel and were not unexpected, even in healthy volunteers. Three bleeding events were potentially related to drug effects on coagulation: two cases of moderate hematoma (one subject given clopidogrel alone; one subject receiving clopidogrel + dabigatran) and one case of moderate hematuria (subject received clopidogrel + dabigatran and subsequently discontinued treatment). However, there was no correlation between the individuals’ PK data and these bleeding events. Although these results are not conclusive, dabigatran etexilate in this study did not seem to cause any safety issues, whether given alone or in combination with clopidogrel.

The study was designed as a phase I study in healthy volunteers and aimed to clarify any PK/PD interaction between clopidogrel and dabigatran etexilate. This type of study does not allow any conclusions to be drawn with respect to the clinical effects of long-term use of the combination in atrial fibrillation patients. However, dabigatran etexilate was administered at the therapeutic dose of 150 mg bid, and clopidogrel was administered at dosages used in clinical practice. The designs of the pilot and two main parts of the study were purposefully chosen. The pilot study (part 1) had a fixed-sequence design (multiple doses of dabigatran 150 mg followed by a single dose of clopidogrel) for safety reasons. In the main study, the crossover design was used as is usually done for drug–drug interaction studies due to its efficiency, i.e., the comparison between treatments based on a comparison within subjects rather than between subjects (part 2). A washout period of 14 days was considered to be sufficient to separate the three treatments, based on the half-life of dabigatran (8–10 h and 14–17 h with single- and multiple-dose administrations, respectively [7]). The combination of dabigatran with a high clopidogrel dose (600 mg) warranted a fixed-sequence design (one drug alone followed by the combination) for safety reasons (part 3).

In summary, steady-state dosing of clopidogrel 75 mg qd (following a 300 mg loading dose) and dabigatran etexilate 150 mg bid demonstrated no effect on dabigatran PK or PD, or its PK/PD relationships (aPTT, ECT, or TT). Similarly, the PK of clopidogrel and its PD effects on the inhibition of platelet aggregation were unchanged by chronic administration of dabigatran etexilate. There may be modest but very transient effects on dabigatran PK (30–35% increase in AUC) when a loading dose of clopidogrel (300 or 600 mg) is administered, with no impact on the PK or PD of clopidogrel.

## Electronic supplementary materials

Below is the link to the electronic supplementary material.

Supplementary Figure 1 Part 2. Geometric mean plasma concentration–time profiles of free and total dabigatran (**a**) and clopidogrel and SR26334 (**b**) after multiple oral administrations of 75 mg clopidogrel once daily (qd) (preceded by a loading dose of 300 mg) with or without coadministration of 150 mg dabigatran etexilate (DE) twice daily (bid) (JPEG 65 kb)

High resolution image (TIFF 1139 kb)

Supplementary Figure 2 Part 3. Geometric mean plasma concentration–time profiles of free and total dabigatran (**a**) and clopidogrel and SR26334 (**b**) after repeated oral administration of 150 mg dabigatran etexilate (DE) twice daily (bid) with or without coadministration of 600 mg clopidogrel (JPEG 64 kb)

High resolution image (TIFF 1089 kb)

ESM 3 (DOCX 26 kb)
