# Supplementary figures and images for: Pharmacokinetic and pharmacodynamic effects of comedication of clopidogrel and dabigatran etexilate in healthy male volunteers
Source: Eur J Clin Pharmacol. 2012 Jul 11;69(3):327–39. doi: 10.1007/s00228-012-1304-8 (PMC3572379; doi:10.1007/s00228-012-1304-8)

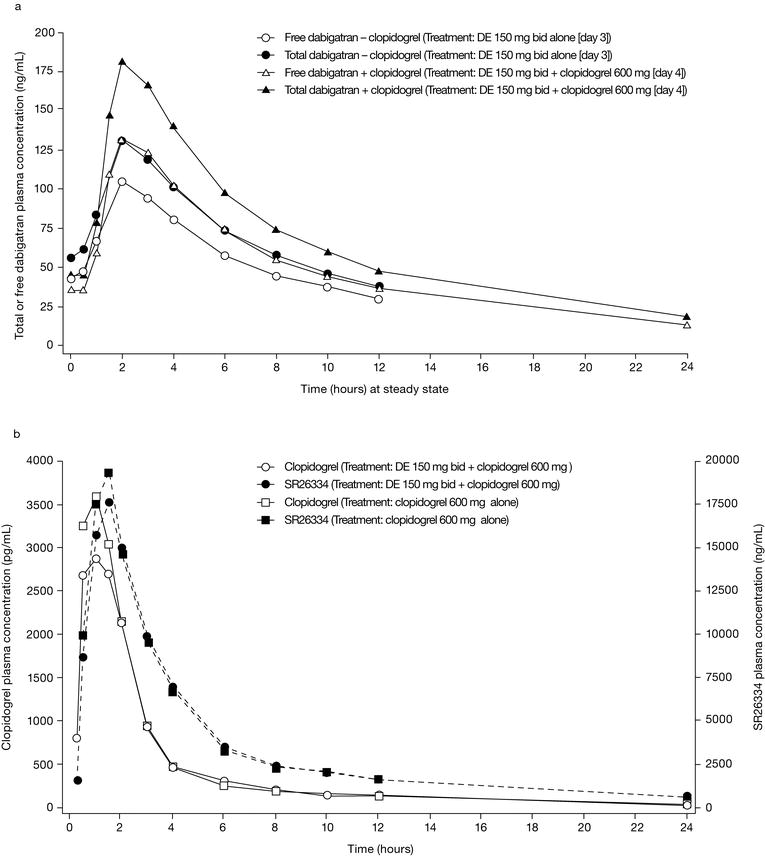

Supplement: Supplementary file 1 — Part 2. Geometric mean plasma concentration–time profiles of free and total dabigatran (a) and clopidogrel and SR26334 (b) after multiple oral administrations of 75 mg clopidogrel once daily (qd) (preceded by a loading dose of 300 mg) with or without coadministration of 150 mg dabigatran etexilate (DE) twice daily (bid) (JPEG 65 kb) [file 228_2012_1304_Fig3_ESM.jpg]

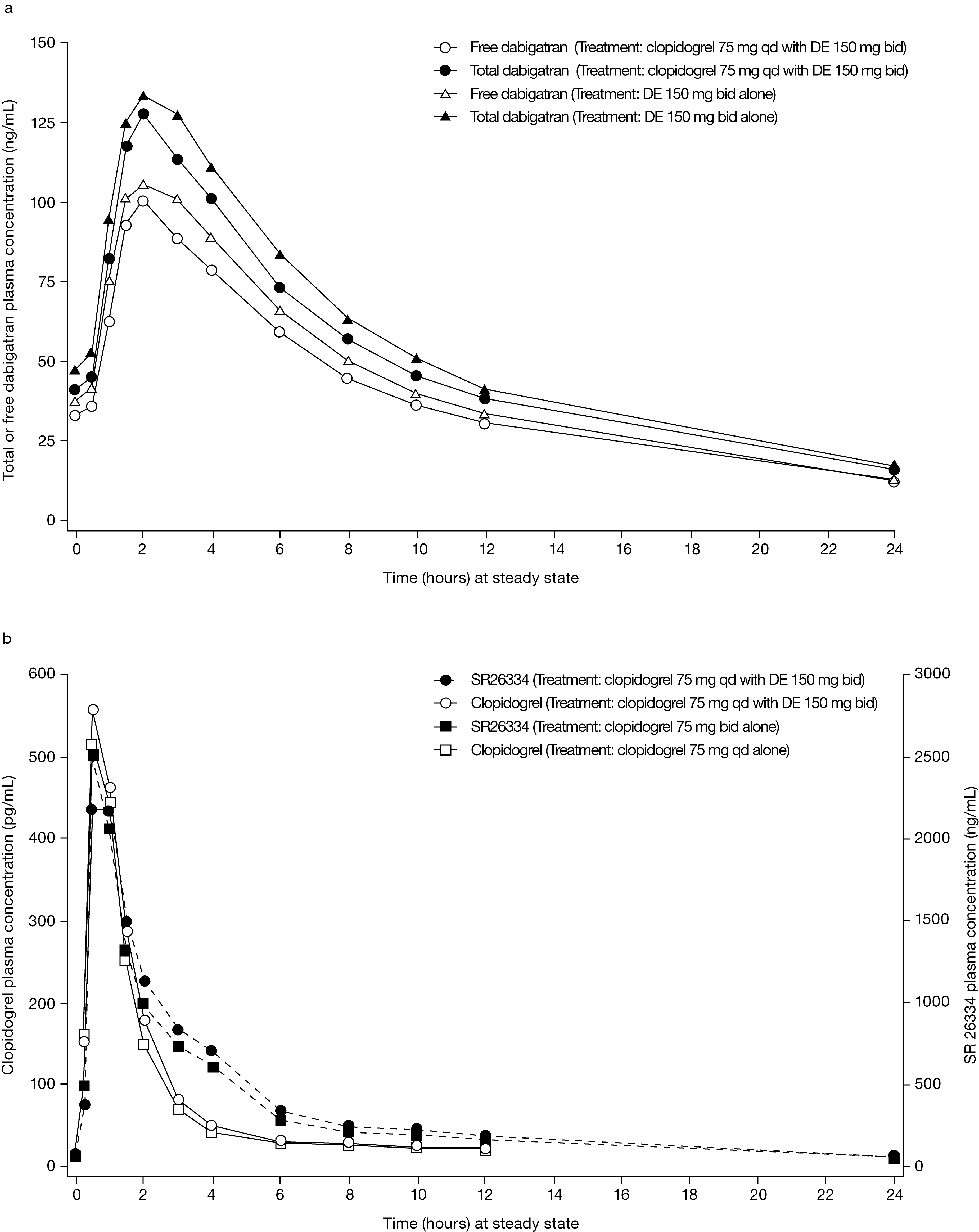

Supplement: Supplementary file 2 — High resolution image (TIFF 1139 kb) [file 228_2012_1304_MOESM1_ESM.tif]

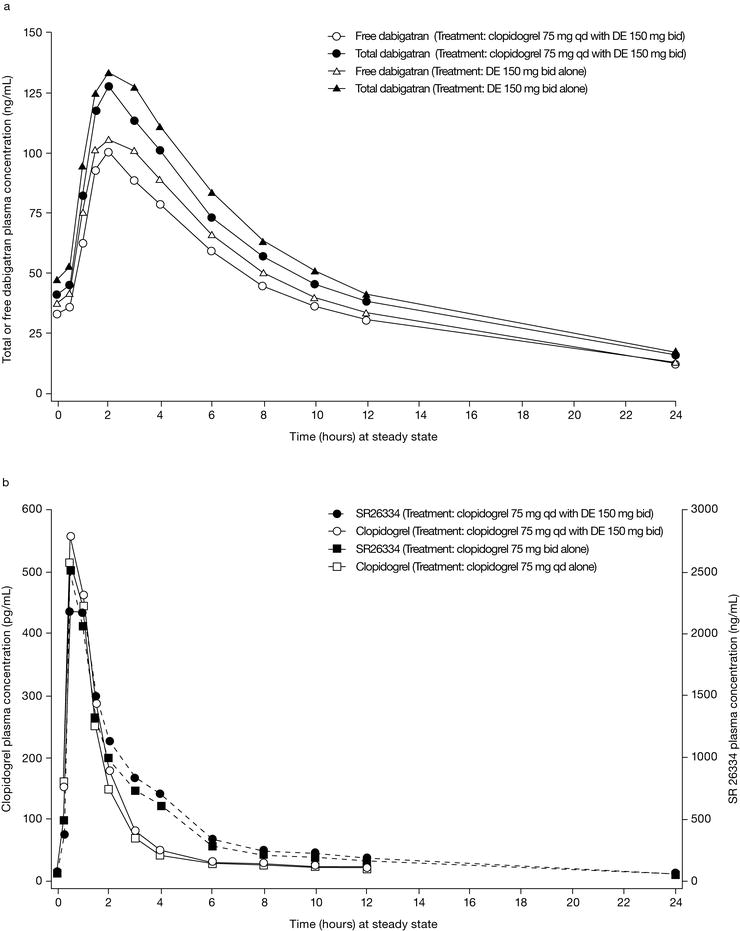

Supplement: Supplementary file 3 — Part 3. Geometric mean plasma concentration–time profiles of free and total dabigatran (a) and clopidogrel and SR26334 (b) after repeated oral administration of 150 mg dabigatran etexilate (DE) twice daily (bid) with or without coadministration of 600 mg clopidogrel (JPEG 64 kb) [file 228_2012_1304_Fig4_ESM.jpg]

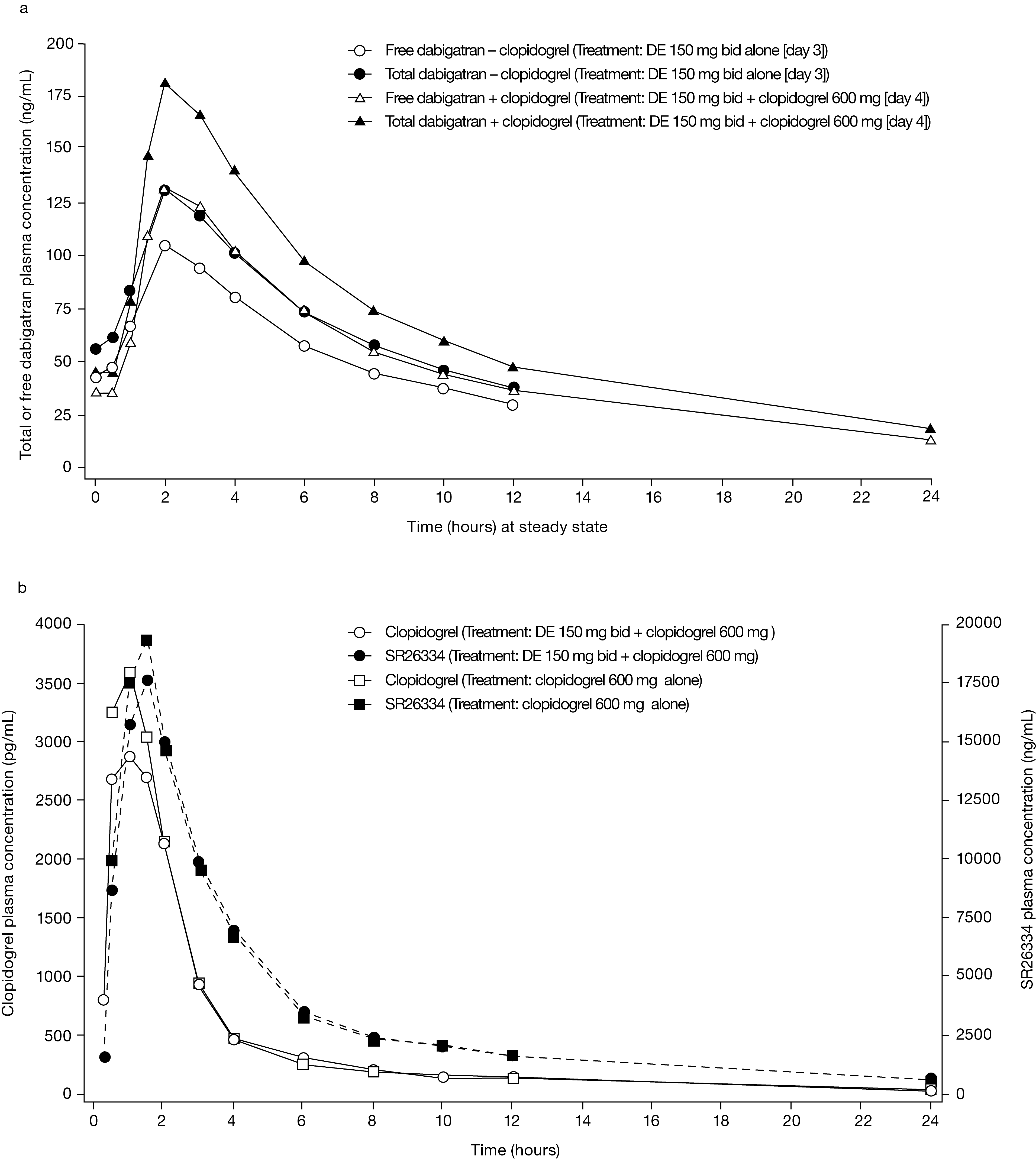

Supplement: Supplementary file 4 — High resolution image (TIFF 1089 kb) [file 228_2012_1304_MOESM2_ESM.tif]
