# Supplementary material for: Pharmacokinetic and pharmacodynamic effects of comedication of clopidogrel and dabigatran etexilate in healthy male volunteers
Source: Eur J Clin Pharmacol. 2012 Jul 11;69(3):327–39. doi: 10.1007/s00228-012-1304-8 (PMC3572379; doi:10.1007/s00228-012-1304-8)
Supplement: Supplementary file 5 — (DOCX 26 kb) [file 228_2012_1304_MOESM3_ESM.docx]

**Supplementary Table 1:** Timings of PK/PD sampling.

**A) Part 2: Clopidogrel with or without dabigatran**

| **Visit** | **Day** | **Planned Time** | **Drug Administration** | **PK Clopidogrel** | **PK, Dabigatran** | **PK Coagulation tests** | **IPA** |
| --- | --- | --- | --- | --- | --- | --- | --- |
| 1 | –21 to –4 |  |  |  |  |  |  |
| 2 or 3 or 4 | –2 | –48:30 |  | X |  | X |  |
|  |  | –48:00 | Clopidogrel 300 mg |  |  |  |  |
|  | –1 | –24:30 |  | X |  |  |  |
|  |  | –24:00 | Clopidogrel 75 mg |  |  |  |  |
|  | 1 | –00:30 |  | X | X |  |  |
|  |  | 00:00 | Clopidogrel 75 mg alone ± dabigatran 150 mg |  |  |  |  |
|  |  | 12:00 | No drug or dabigatran 150 mg |  |  |  |  |
|  | 2 | 23:30 |  | X | X |  |  |
|  |  | 24:00 | Clopidogrel 75 mg alone ± dabigatran 150 mg |  |  |  |  |
|  |  | 36:00 | No drug or dabigatran 150 mg |  |  |  |  |
|  | 3 | 48:00 | Clopidogrel 75 mg alone ± dabigatran 150 mg |  |  |  |  |
|  |  | 48:15 |  | X |  |  |  |
|  |  | 48:30 |  | X | X | X |  |
|  |  | 49:00 |  | X | X | X |  |
|  |  | 50:00 |  | X | X |  |  |
|  |  | 51:00 |  | X | X | X | X |
|  |  | 52:00 |  | X | X |  |  |
|  |  | 53:00 |  | X | X | X | X |
|  |  | 54:00 |  | X | X | X | X |
|  |  | 56:00 |  | X | X | X | X |
|  |  | 58:00 |  | X | X | X |  |
|  |  | 60:00 |  | X | X | X | X |
|  | 4 | 72:00 |  | X | X |  |  |
| 5 | 5 |  |  |  |  |  |  |

**B) Part 2: Dabigatran without clopidogrel**

| **Visit** | **Day** | **Planned Time** | **Drug Administration** | **PK, Dabigatran** | **PK Coagulation tests** |
| --- | --- | --- | --- | --- | --- |
| 1 | –21 to –4 |  |  |  |  |
| 2 or 3 or 4 | 1 | –00:30 |  | X | X |
|  |  | 00:00 | Dabigatran 150 mg |  |  |
|  |  | 12:00 | Dabigatran 150 mg |  |  |
|  | 2 | 23:30 |  | X |  |
|  |  | 24:00 | Dabigatran 150 mg |  |  |
|  |  | 36:00 | Dabigatran 150 mg |  |  |
|  | 3 | 47:30 |  | X | X |
|  |  | 48:00 | Dabigatran 150 mg |  |  |
|  |  | 48:30 |  | X | X |
|  |  | 49:00 |  | X | X |
|  |  | 49:30 |  | X |  |
|  |  | 50:00 |  | X | X |
|  |  | 51:00 |  | X |  |
|  |  | 52:00 |  | X | X |
|  |  | 54:00 |  | X | X |
|  |  | 56:00 |  | X | X |
|  |  | 58:00 |  | X | X |
|  |  | 60:00 |  | X | X |
|  | 4 | 72:00 |  | X |  |
| 5 | 5 | 96:00 |  |  |  |

**C) Part 3: Fixed sequence clopidogrel alone and with or without steady state dabigatran**

| **Visit** | **Day** | **Planned Time** | **Drug Administration** | **PK, Clopidogrel** | **PK, Dabigatran** | **PK Coagulation tests** | **IPA** |
| --- | --- | --- | --- | --- | --- | --- | --- |
| 1 | –21 to –4 |  |  |  |  |  |  |
| 2 | 1 | –00:30 |  | X |  | X | X |
|  |  | 00:00 | Clopidogrel 600 mg |  |  |  |  |
|  |  | 00:30 |  |  |  |  |  |
|  |  | 01:00 |  |  |  |  |  |
|  |  | 01:30 |  |  |  |  |  |
|  |  | 02:00 |  |  |  |  |  |
|  |  | 03:00 |  |  |  |  |  |
|  |  | 04:00 |  |  |  |  |  |
|  |  | 06:00 |  |  |  |  |  |
|  |  | 08:00 |  |  |  |  |  |
|  |  | 12:00 |  |  |  |  |  |
|  | 2 | 24:00 |  |  |  |  |  |
| 3 | 1 | –00:30 |  |  | X | X | X |
|  |  | 00:00 | Dabigatran 150 mg |  |  |  |  |
|  |  | 12:00 | Dabigatran 150 mg |  |  |  |  |
|  | 2 | 23:30 |  |  | X |  |  |
|  |  | 24:00 | Dabigatran 150 mg |  |  |  |  |
|  |  | 36:00 | Dabigatran 150 mg |  |  |  |  |
|  | 3 | 47:30 |  |  | X | X |  |
|  |  | 48:00 | Dabigatran 150 mg |  |  |  |  |
|  |  | 48:30 |  |  | X | X |  |
|  |  | 49:00 |  |  | X | X |  |
|  |  | 50:00 |  |  | X | X |  |
|  |  | 51:00 |  |  | X |  |  |
|  |  | 52:00 |  |  | X | X |  |
|  |  | 54:00 |  |  | X | X |  |
|  |  | 56:00 |  |  | X | X |  |
|  |  | 58:00 |  |  | X | X |  |
|  |  | 59:55 |  |  | X | X |  |
|  |  | 60:00 | Dabigatran 150 mg |  |  |  |  |
|  | 4 | 71:30 |  | X | X | X | X |
|  |  | 72:00 | Dabigatran + Clopidogrel 600 mg |  |  |  |  |
|  |  | 72:15 |  | X |  |  |  |
|  |  | 72:30 |  | X | X | X |  |
|  |  | 73:00 |  | X | X |  |  |
|  |  | 74:00 |  | X | X | X | X |
|  |  | 75:00 |  | X | X |  |  |
|  |  | 76:00 |  | X | X | X | X |
|  |  | 78:00 |  | X | X | X | X |
|  |  | 80:00 |  | X | X | X | X |
|  |  | 82:00 |  | X | X | X | X |
|  |  | 84:00 |  | X | X | X | X |
|  | 5 | 96:00 |  | X | X | X | X |
|  | 6 | 120:00 |  |  |  |  | X |
| 4 |  |  |  |  |  |  |  |
